# Collectanea Medica, Consisting of Anecdotes, Facts, Extracts, Illustrations, Queries, Suggestions, &c.

**Published:** 1816-04

**Authors:** 


### COLLECTANEA MEDICA,

CONSISTING OF

### ANECDOTES, FACTS, EXTRACTS, ILLUSTRATIONS, QUERIES, SUGGESTIONS, &c.

Quicquid agunt medici,

Nostri farrago libelli.

[The following paper is so important as to speak for itself throughout: we have, therefore, preferred giving the whole of it in this place, to reserving it for our Critical Analysis.]

*Some Cases, illustrative of the Pathology of the Brain.* By RICHARD POWELL, M.D. Fellow of the Royal College of Physicians. *(From the last vol. of Medical Transactions.)*

THE pathology of the brain and nervous system appears even at present to be more defective than other branches of medical science; and most practitioners must have felt cause to lament their previous uncertainty, with respect to the altered condition of these parts, when the real nature of the diseases has been ascertained by anatomical investigation. If we further compare the actual symptoms of various affections of the brain, with those by which they are characterized in systems of nosology, we may, perhaps, be led to wish that the whole subject should be new-modelled. Inflammation of its membranes, for examples, is by no means unfrequent, whilst we rarely find it accompanied by the symptoms, which should designate phrenitis; for, as far as my opportunities of observation have gone, they have been referable rather to oppression of nervous power, than to increased activity of the circulating system. On the other hand, patients have often been destroyed under symptoms of disease of the brain, and yet that altered structure of the parts, which may have been strongly inferred from the symptoms, has not been found to exist.

It is by the accumulation of facts, and by the connection of symptoms with organic alteration of structure, that we can best attempt to promote our knowledge of this class of diseases, notwithstanding there may be, and probably will for ever remain, a vast number of important cases, for the explanation of which morbid anatomy will be found wholly insufficient. In a former Paper, I endeavoured to point out this insufficiency, in one variety of paralytic affection: in the present, I intend to record some cases which have fallen under my own observation, and which were unfortunate in their termination, for the purpose of connecting the morbid appearances, or the absence of them, with the symptoms which existed during the life of the patient. Although neither the dissections nor their histories may be new, nevertheless I should hope that the combination of them may offer some points deserving the attention of the College. Still I am fully aware of their their deficiencies, both as to the histories of symptoms, and the anatomical detail of appearances. With respect to the former of these, I believe that most practitioners, who note down the circumstances of disease, must, like me, when they have afterwards attempted to revise and to collate their observations, have found in how many points their notes were imperfect, and have regretted the many illustrative particulars which have been overlooked at the moment, and cannot afterwards be recovered. As to the latter, a confident hope may be expressed that the cases of diseased brain, which shall hereafter be recorded, will be more minute in their detail, and more precise in their descriptions of local situation; and that the dissections recently introduced into this country, by Dr. Spurzheim, of Vienna, will not only improve our anatomical knowledge, but ultimately lead also to great improvements in the pathology of this most important organ.

Case I.—A young lady, aged 17, had, on February 28, attended the service of the church, and afterwards walked in Hyde Park, in the apparent enjoyment of her usual health and spirits. In the evening, she felt herself unwell, and complained of general soreness, as from severe cold. Through the following day, March 1, she was observed to be very heavy, and much disposed to sleep; and, when I first saw her, about the middle of the 3d, she had lain from the preceding morning in a state of perfect stupor and insensibility, interrupted only by occasional attacks of strong and general muscular convulsions. Such a fit took place during my visit. She could not be roused to sensibility, and the convulsive motions appeared to be equally strong on each side of the body; the pupils were much dilated, and only very slightly influenced by any application of strong light, which she seemed, nevertheless, to avoid, closing her eye-lids by a sort of voluntary effort, as if it were offensive; and when a liquid was held in her mouth for some time, it produced an effort by which a small portion of it passed into the stomach. The general character of her countenance was stern and contracted. Leeches and blisters had been applied, and motions had been obtained, but she passed them without consciousness. She was stated not to have been regular in menstruation for some time before, and to have recently used various lotions for the purpose of repelling a slight eruption upon her hands. I directed the application of a cold wash, with vinegar, to her head, and of sinapisms to the feet; and that, if possible, a solution of sulphate of zinc should be given, so as to produce vomiting. She seemed to feel nausea, and to heave a little from what was swallowed, but vomiting did not take place. It was thought, in the evening, that she had given signs of increased irritability and consciousness, particularly by the manner in which she rejected what was put into her mouth, which seemed to depend upon voluntary effort; and her convulsions had also been less frequent. Towards morning, however, the convulsive attacks became more violent, and almost constant, and she appeared to suffer considerable pain; it was thought that the signs of this were more evident evident when the abdomen was pressed upon, yet her bowels had been fully evacuated on the preceding day. The pupils were largely dilated and wholly insensible, the skin was losing its temperature, the pulse became small and countless, and she died in the afternoon. The brain was, on the following day, most minutely and accurately examined by Mr. Young, and no appearance of disease whatever, no alteration of structure was discoverable, nor was there any thing wrong in the abdominal viscera.

Case II.—The servant of a gentleman, in my neighbourhood, had attended his master, on horseback, and returned home to dine as usual. It was afterwards recollected, that he had complained of some slight head-ach before breakfast, but not at any subsequent period of the day. He conversed throughout the evening, with an old female servant, in his ordinary manner; and whilst he was occupied in writing out some accounts, he dropped suddenly from his chair, and expired without a single groan, or apparent struggle of any kind.

I was not present when the head was examined on the following morning, but I understood that a considerable quantity of blood was found to have been effused into the ventricles of the brain.

Case III.—A gentleman brought his son, aged 8, from a school in the neighbourhood of London, on account of an attack of head-ach, which he had suffered in the preceding evening, and a sort of convulsive fit, which he was stated to have had in the course of the night. It appeared tolerably certain that he had received no injury, and his illness was not thought to be of any great importance. On his road home, he was attacked in the carriage by convulsions, which were followed by stupor. He arrived about three, p. m. and I saw him at four. The livid look about his countenance, the foaming mouth, the general insensibility and occasional violent convulsions, and the oppressed irregularity of the pulse, indicated the existence of such a degree of pressure upon the brain as seemed very unlikely to yield to any mode of treatment. The temporal artery was opened with little effect, and motions were obtained, but he died about eight o'clock.

The surgeon who examined the head stated that the brain itself appeared unusually large, and the vessels of the dura and pia mater were enormously loaded with blood; an accumulation of aqueous fluid had also taken place between the membranes; about two fluid ounces of water, slightly tinged with blood, were found within the two lateral ventricles, and the vessels of the plexus choroides seemed to have given way and produced this tint; the thoracic and abdominal viscera were all perfectly healthy, and the intestines contained very little feculent matter.

Case IV.—A gentleman became suddenly insane, after an attack of severe diarrhœa of some standing. His imagination was more than commonly active, and his ideas were exceedingly elevated; but though his premises were false, his conduct and inferences founded upon them were sufficiently correct. He was very impatient of restraint, and often started out into fits of greater violence.

violence. I lost sight of him for about two years, which he had passed in confinement, and under the treatment of physicians of the highest and best practical experience. When I next saw him, he had sunk into a state of fatuity, and passed both his urine and stools involuntarily. He had no partial loss of power, and no other sign of paralytic affection; but, at uncertain intervals, he was seized by convulsive fits, under which the left half of the body suffered more considerably than the right, and at last, under a very severe attack of the same sort, he died.

When the head was examined, an adventitious membrane was found under the dura mater, which extended over the right hemisphere of the brain, and upon the falx of the same side, and reached the base of the skull. It was somewhat adherent to the dura mater, but very easily separated from it, and from the arachnoid coat beneath. It was highly vascular, of firm texture, and of about the thickness of three sheets of writing paper, but, in proportion as it reached the basis of the brain, it became thinner, and was lost. The arachnoid coat itself, of the same side, was more vascular and thicker than natural, and under it a quantity of gelatinous fluid was collected. The membranes which covered the opposite hemisphere were more vascular than natural, but there was no similar appearance, on that side, of an adventitious membrane. The ventricles contain about two fluid ounces of aqueous fluid.

Case V.—A young gentleman, aged 16, applied to me, on the 9th of November, on account of an eruption, with an acrid discharge, behind the right ear. I understood that he had become deaf five years before, after scarlatina, but that no discharge had, at that time, taken place from the ear; and that, in the following year, after measles, an abscess had formed in the right ear, with considerable pain, and had burst. When I next saw him, on the 17th of November, I learned that he had suffered a sudden attack of very severe pain, in the same ear, on the 14th, which had wholly disturbed his rest; and, as he stated, resembled very closely that which he had before undergone when the abscess formed in it. There was an appearance of pus in the bottom of the ear, some of which, when wiped away, was yellowish, uniform, and not offensive to the smell. The pulse was 60, firm and equable; the tongue was white and coated; but the bowels were regular in their action, and the stools natural. On account of the intensity of the pain, twenty-five drops of the tincture of opium were given at night, by which he was entirely relieved as long as its influence lasted, but as this wore away, the pain again increased with great violence, and the night of the 19th was a very restless and disturbed one. The external inflammation and discharge had diminished, but not wholly subsided; and within the ear there appeared to be a projecting tumour, confining some pus beyond it; but still there was a considerable portion of pus collected upon a poultice, which had been applied over the ear, and this was also more offensive than it had before been. Eight drops of the tincture of opium were given every every six hours, for the purpose of keeping up a uniform effect; and he expressed much satisfaction at the relief he received from it, stating, that though the pain was still severe, it was rendered bearable by the medicine. He distinctly described it as existing within the ear without spreading to the adjacent parts. The pupils of the eyes were equally and fully sensible to light, which was not in the least offensive, and his mind and senses were natural and perfect. His tongue had cleaned; his pulse continued at 60, firm, and regular; and I also noticed a marked pulsation in his neck, a symptom to which I am especially attentive, on account of its uniform and almost characteristic connection with some diseases of the heart. On the 22d the opiate was omitted; but on the 23d it was necessarily repeated, on account of the violence of the pain, which was again considerably checked by its use, though his sufferings continued to be very great. There was nothing like delirium or coma: he dozed, indeed, a great deal, but he was easily roused, and answered questions distinctly and correctly, and appeared to possess his perfect senses in every respect. He turned voluntarily from his right to his left side in bed, for the purpose of directing the light into his ear, that I might examine it. When I did so, I saw some thick pus, but not much in quantity, lodged in the bottom of the ear, and both this portion, and that which had collected on the poultice, had a earthy and offensive smell, which led to a suspicion that the bone might be affected. The skin was warm, moist, and natural; the pulse 72, and firm; and the excretions sufficient and healthy. In the course of the same night, the pain increased to a most violent degree, his powers sank rapidly, and he died about six in the morning.

When the head was examined, the structure of the dura mater was healthy and natural; but, beneath this membrane, the whole superior surface of the right hemisphere was covered with a layer of coagulable lymph and pus, a considerable quantity of which was also collected between the posterior lobe of the cerebrum and the tentorium. The whole quantity, collected, as well as it could be, by a sponge, exceeded three fluid ounces. The vessels of the substance of the brain were not more numerous or loaded than usual, and the brain itself was healthy in every part. In the base of the skull, the dura mater adhered to the bone, except in one part, of about half an inch diameter, just over the petrous portion of the temporal bone, where it was black and sloughy; the subjacent portion of the bone itself was carious, black, and crumbling, and contained fetid pus. The auditory nerve looked healthy.

Case VI.—J. M., aged 70, was admitted under my care into St. Bartholomew's Hospital, September 1, 1814, labouring under a constant general convulsive affection of the left side, which had attacked him suddenly about a fortnight before, and continued until the time of his admission. At first, however, it was much slighter in its degree, but it had grown gradually more violent, and then seemed to constitute a very severe case of hemiplegic chorea. The right side of the body also, though not convulsed, was much

 weakweaker than it ought; and he stated that it had constantly been so for thirty-four years, in consequence of a typhus fever. His general health was in other respects good, and his intellectual powers were free and perfect. After the exhibition of a purgative medicine, the nitrate of silver was directed in doses of one grain, and in the form of a pill, every four hours, and at the end of a week was increased to two grains. Within the following week he had acquired a greater command over the affected side, and expressed himself as mending very fast. He slept sufficiently; and during sleep was wholly free from the convulsive motions; his bowels were regular in their action, and his motions were figured and natural. The dose of the medicine was then further increased to three grains every four hours, and, as on the following day he complained of flatulence, which he ascribed to its effects, the infusion of quassia and tincture of ginger were given at the same time. He seemed to be considerably improved on the 21st, but on the 22d, without the operation of any ostensible cause, he was suddenly seized with hemiplegia of the left side, with loss of power of speech, and stertorous breathing; and his pulse became preternaturally slow, full, and hard. After this attack, the right hand and arm became constantly and tremulously convulsed, but much more weakly so than the left had been. Large and repeated loss of blood, from the arm, produced much temporary benefit; and after it, on the 24th, he articulated distinctly, and was able to answer questions with a clear comprehension of their import, and his pulse became soft and natural, and rose to 84. After this time, he became gradually more and more comatose; he did not express his sensations of wanting food, but took any nourishment which was offered him freely, and even ravenously. From October the 1st, he passed his stools and urine voluntarily, his senses and general powers failed him, and after having lain for some days in a state of complete insensibility, he died on the 18th.

Mr. Stanley examined the head on the following morning. There was general opacity of the tunica arachnoidea and pia mater, and a very considerable effusion of aqueous fluid into the cellular texture of the latter. The ventricles were distended also by a transparent aqueous fluid, amounting, in quantity, at least to three fluid ounces; and there was a similar collection between the membranes at the base of the brain, so that the aggregate amounted to about half a pint. In the fore part of the anterior lobe of each hemisphere, a destruction of the substance of the brain had taken place, apparently from ulceration, for the surface of the diseased part presented an irregular excavated appearance, with a thin layer of curdled matter deposited on it. This diseased appearance occupied a space in the right hemisphere of about two inches in breadth, and as much in length; but in the left hemisphere it was less extensive.

Case VII.—A. P., aged 23, was under my care, in St. Bartholomew's Hospital, for twenty-seven days before her dissolution, which took place January 31, 1810. It appeared that her illness had commenced about three months before, at the time of menstruation, struation, in consequence of a violent cold; and that the discharge had not again taken place. Since that period she had suffered frequent hysterical fits, with much flatulence and rising in the throat; but at the time of her admission, her complaint was wholly referred to the head, where she complained of excessive pain. It seemed, however, to vary considerably in its violence; at some times it was insupportable, and at others was described rather to be a dull and heavy weight, than actual pain. Her sight was imperfect and misty. Her eyes were full and starting, and their pupils dilated and indolent; but they contracted when a strong light was applied. The repeated application of leeches, with blisters, and the frequent use of purgative medicines, produced a temporary alleviation of her sufferings. In the night of the 12th of January, she was attacked, without any previous notice, with a sort of fit, under which she was described to have been totally insensible for about an hour, whilst her head and neck were drawn backwards, and rendered stiff and immoveable. Her speech was wholly lost on the following morning, but, in the course of the two following days, she gradually acquired the power of expressing herself, and still complained of violent pain in the head. Her sight became less and less perfect; the pupils, which at first had been dilated and indolent, ceased to be at all affected by light; their loss of susceptibility, however, was not uniform, for they seemed to vary in this respect from day to day; but, after the 20th, all power of vision was entirely gone. She passed her nights throughout with a diminution rather than increase of inconvenience, and slept moderately well. The powers of her mind were not at any time disturbed or impaired by her disease. The pulse was always weak, small, and slow, and was sometimes as low in number as 54. The tongue was usually furred and dry, and she had some attacks of nausea and vomiting, but they were only slight: she always felt disposed to take her food; and her bowels, though costive if unattended to, were nevertheless easily regulated by mild opening medicines. On the 19th she had another attack of fit, which began with a cold shivering, after which she sank into a state of complete insensibility, and suffered no convulsive motion of the limbs during it. From this attack she never recovered her powers to any degree, and after the 22d she fell into a state of permanent stupor, from which, for the two next days, she could be roused so as to answer questions without any difficulty, and at such times she always complained of the violence of the pain in her head. Her strength gradually diminished until her death on the 31st.

On examining the head, all the membranes appeared to be much more loaded with blood than natural. The convolutions of the brain were rather flattened, and in three places, upon the surface of the right hemisphere, there were hardened spots of about an inch diameter. These constituted a part of the same number of considerable tubercles which extended into the medullary substance of the brain. One other tubercle was also more deeply and wholly embedded in the medullary substance of the cerebrum. There was one similar spot upon the left hemisphere, but it was not so large, nor did it extend so deeply. On pressing down the brain with an equal force, it was evident that the right hemisphere was larger than the left, and must have encroached upon it considerably, and moved it from its usual station. The ventricles contained more aqueous fluid than natural. The cerebellum and tuberculum annulare exhibited only those appearances which belong to their usual structure.

Case VIII.—For the detail of the following case I am obliged to Dr. Warren, from whose notes it is furnished: I attended the subject of it occasionally, in consultation with him, during the last three weeks of his life.

Mr. L. was by birth a Peruvian, and had served several years in the Spanish army in Europe. During the latter part of his time he had been actively employed, and suffered great anxiety of mind from various causes. About two years before his death, he was attacked by very severe pains in his head, for which no permanent relief was obtained; but by degrees they subsided, leaving behind them a constant sensation of tightness across the middle of the forehead, and a feeling of want of room within the skull. Nearly six or eight months after the first attack, while sitting in one of the theatres at Paris, he was sensible of a sudden diminution of the sight of the right eye; and this defect increased until the power of vision on that side was completely lost. The sight of the left eye then became impaired in the same manner; and, as the affection of this eye advanced, the other recovered a small power of vision, but in the progress of the disorder he became completely blind. In this state, among other remedies, emetics were used, and the effect of them was remarkable. In the act of vomiting, the power of vision was suddenly restored to the right eye, with a sensation as if a flash of lightning had taken place; but it remained only for the short space of an hour, the clear vision gradually subsiding to a glimmering of light, and at last becoming extinguished. In this state of blindness he remained more than twelve months, when he applied to Dr. Warren for the relief of some stomach complaints. The pupils were at this time dilated to their utmost extent, and the eyes were totally insensible to light. He had been for some time under the care of an eminent oculist, on account of the amaurosis. The complaints, for which he required medical assistance, were weakness of digestion, uneasiness in the epigastric region, want of appetite, and frequent disposition to vomit. The bowels were inactive, but not very costive; urine was passed in considerable quantities; the pulse was free and natural; the tongue of a pale colour. At this time he did not complain of pain in his head, but of the tightness of the forehead and upper part of the skull, before related. He was sluggish and indolent; but his intellects were clear, his mind vigorous, and actively engaged in the politics of his native country. A partial relief of his stomach complaints was obtained by medicine, but the disposition to vomit continued, and by degrees he became weaker and very costive.

His

His sight having been temporarily recovered in Paris by the use of emetics, it was advised that they should be tried again. After the use of the second emetic, the pupils of the eyes recovered the power of dilating and contracting on exposure to light, and preserved it till death; but the power of vision was not restored. Two days afterwards he was threatened with a fit of apoplexy, from which state he was relieved by cupping, and the application of a blister. His strength declined rapidly after this attack; he became very sleepy; he next lost the power of distinct articulation, but preserved the faculties of his mind; these he retained till within a few hours of his death, with sufficient distinctness to give his assent or dissent to any proposition that was made to him; but he did not appear capable of following any continued train of thought. During the whole period of his illness, the sense of hearing had been very acute, and to the last day of his life enabled him to recognise, with great exactness, the voices of persons with whom he was acquainted. After death, the head was examined by Mr. Brodie. The tunica arachnoides was considerable thickened where it covers the upper part of the cerebrum. The ventricles of the brain contained about four times the usual quantity of water, with a small quantity of matter floating in it. The pituitary gland, which is situated below the optic nerve, was converted into a pulpy structure, about five or six times the usual bulk of the gland; and the sella turcica, which contains it, had become enlarged in the same proportion. To the upper part of the gland was connected a tumour of an oval form, and of the size of a hen's egg, containing a thick purulent fluid; it was situated under the middle lobe of the cerebrum, and interposed between the optic nerves, which were, in consequence, much separated from each other. The tumour occupied the place of the infundibulum, extending into the third ventricle; and there was every reason to believe, that the pus in the ventricles had passed through a minute aperture in that portion of the tumour which was contiguous to them, although, from the circumstances of the dissection, the opening could not be distinctly demonstrated. The fibres of the optic nerve were seen expanded, and almost destroyed, on the side of the tumour. The vessels of the brain generally were more turgid than usual. The plexus choroides contained some small concretions of earthy matter, and was somewhat thickened.

Case IX.—I saw, in consultation, on August 10, 1814, a gentleman, aged 29, who first complained of head-ach, after very slight exercise in an exceedingly hot day, July 28, but whose symptoms had excited no alarm even in the mind of an able and anxious medical friend, until the 14th of August, when the pain increased, and he began to wander in his mind, with much unusual heaviness and stupor, in which state he had since continued. He was unable to answer, and did not seem to comprehend any questions which were put to him. He tossed his head about occasionally as if from pain, but he expressed none. The pupils were much dilated, and were not affected by a strong light. His articulation was very imperfect perfect and indistinct, and not regulated by any consciousness of what he uttered; but in this respect his powers were said to vary, and sometimes he was more active, and easily roused. He had previously laboured under an affection of a testicle, which had suppurated and returned to its natural bulk, and certainly did not depend upon any venereal cause. It was recollected by his friends, that he had occasionally laboured under attacks of head-ach, and that such a one came on about three months before, with a considerable throbbing sensation. The pulse was 100, and oppressed. He had been blistered and purged; but leeches had not been applied from the experience of their tendency to produce erysipelas.*

Sixteen fluid ounces of blood were taken from the temporal artery, and ten grains of submuriate of mercury were ordered to be given every second hour, until stools were obtained. In the course of the night he passed, involuntarily, abundant fæcal black stools; and seemed afterwards to be more roused, and more readily alive to external objects. The sufficiency of his muscular powers were also evinced by the way in which he turned in his bed; and his pulse was 120, more free and soft. His head was next blistered; and his speech and his mind became clearer and more collected, for he comprehended questions which were put to him, and answered them shortly but rationally. His sight also was perfect, for he knew and named some of his friends around him. He put forth his tongue, which was white, and thickly coated; and I was able to examine his mouth, which was covered with thick aphthæ. Hiccough became a most troublesome symptom, and followed every thing he took into his stomach, either as medicine or nourishment, and it was with difficulty kept under by small quantities of tinctura opii. From the 25th he began to sink rapidly, though he continued sensible. His right eye-lid dropped so as half to close the eye, the pupil of which was more dilated than that of the left, and was insensible to light, whilst the latter contracted. He did not speak, or seem to possess any consciousness, on the next day; and, on the 27th, his right side had become paralytic, and he died.

The head was examined on the following day. The blood-vessels of the brain were very turgid, and the ventricles considerably enlarged and distended, by at least four fluid ounces of aqueous fluid. The convolutions of the brain were flattened, and its substance throughout was remarkably soft. There was a layer of yellow substance covering the pons varolii, and enveloping the

* I have not sufficient experience to affirm the fact, but I have reason to believe that the variety of leech with an olive-coloured uniform inferior surface, which is brought from Portugal, and has of late been much employed in this country, is by its bite more frequently productive of erysipelas than the usual one, whose inferior surface is variegated with black and yellow, and which used to be considered as the regular Hirudo medicinalis.

origin origin of the optic and other nerves: it resembled the coagulable part of the blood, and seemed to be formed by an effusion of it into the cellular texture of the pia mater. At the anterior part of the middle lobe of the brain, the pia mater was much thickened, and contained a quantity of small white tubercles, not much exceeding a large pin's head in bulk; and a similar kind of tubercles was also found, but in smaller numbers, over nearly the whole of the same membrane, particularly in its processes, which dip between the convolutions of the brain. These tubercles projected also from the inner side of the membrane, the outside of which was smooth and uniform.

Case X.—I saw a gentleman, in consultation, on October 17, who had then suffered from violent pain in the head for about a fortnight, during which time bleeding and purging had been actively employed, and with considerable temporary relief. A recurrence of the affection, however, had taken place with equal violence, and the same means seemed, on repetition, to have almost lost their effect. This pain exacerbated by paroxysms, but not at any regular periods: when under their influence he felt exceedingly depressed, but on their abatement resumed his usual spirits. The pupils were sensible to light, and vision was perfect, but under the increased severity of the pain became double; and it varied a good deal in these respects, at different times, during the progress of the disease. He described himself also at one time to have felt a considerable degree of muscular twitching, and numbness on the left side of the body. The pulse was 54. The tongue was dry and coated; and the bowels were torpid in their action. Twenty fluid ounces of blood were immediately taken from the temporal artery, which much relieved him for a few hours; but the pain recurred, and at the same time the cut artery happened to burst open: advantage was therefore taken of the circumstance to allow the discharge of ten fluid ounces more. He passed the succeeding night without pain, and slept well, and seemed much refreshed in the morning. The pulse was then 96. The tongue was cleaner and more moist; abundant stools had been obtained, and he felt a disposition to take food. Under the apprehension, however, of disease within the head, a perpetual blister was directed to be established upon it, and one grain of the submuriate of mercury to be taken thrice daily. He went on well until the 22d, when the pain again attacked him, and was again overcome by the loss of eighteen fluid ounces of blood from the temporal artery. The exacerbation was, in this instance, distinct and sudden, and it afterwards assumed the same character of remission and accession in a more marked manner. The pulse again sank to 54, and the bowels became more torpid and difficultly acted upon. In addition to the submuriate, we judged it proper to secure the full effect of mercury by rubbing in a drachm of the ointment every six hours, until an affection of the mouth should be produced, and this did take place on the 27th. A pill, containing four grains of aloë was also given every six hours, by which a full and free action of the bowels was established. In the night of the 23d, he had another severe attack, which lasted four hours before it began to abate; and it was not entirely gone at the end of five more, when it again exacerbated in a very considerable but not quite so violent a degree; and he then referred the pain more definitely to the back part of the head than he had done before, for he had described it as general, and rather worse across the forehead. For some days following, the attacks were less violent, but his vision remained permanently double. The pulse continued also pretty regularly at 78.

I was again desired to see him on November 16th, and I found that he had continued free from any attack from October 26, to the preceding evening, during the greater part of which time he had been under the influence of mercury. On the 14th, he had attempted to walk out, and felt himself worse after the exertion, to which indeed he ascribed the present recurrence. The paroxysm appeared to have been extremely severe, but circumscribed in its extent, and confined to the forehead; during it there also seemed to have been violent spasmodic exertion of the muscles connected with the head. He did not, however, describe it as involuntary, but as a sort of effort from which he sought relief. His sight was distinct, his mind and senses were perfect, and his bowels acted regularly; but he appeared to be much weakened, and to have become much more irritable. Ten grains of camphor were ordered every six hours, and he thought that he slept better on the following night, and that the attacks were mitigated in their severity by its use: this, however, did not continue, and, on the 19th, the complaint seemed to have assumed so much of the character of distinct paroxysms, and the intervals to have a greater, or rather it might be said, a perfect freedom from complaint; that, although there were several of these during the space of twenty-four hours, I judged it proper to try the effect of cinchona in substance, and given freely. During these intervals the pulse was 72, and the bowels were regular; under this plan the attacks were thought to be less severe, but they were not less frequent, and they seemed also not to consist of pain alone, as at first, or to excite voluntary muscular effort, as they were before described to do, but to attack suddenly, and to be accompanied by muscular convulsions, chiefly of the right side; through these attacks, however, he also retained his senses. Other modes of treatment, which were afterwards adopted, and among these a seton in the neck, exerted very little influence over the complaint in its subsequent progress. The paroxysms themselves varied a good deal: they were uncertain in their recurrence and degree, and at some times were marked by pain alone, at others by convulsions of the right side; but, upon the whole, they were considered as having abated much of their severity. He lay in his bed on either side indiscriminately, and was usually easiest when his head was kept drawn backwards. On the 26th he was able to sit up for two hours, and he did the same on the the 30th, when he also expressed himself to be much more comfortable in his own feelings; but in the same night, after a comparatively slight convulsion, he died.

The head was examined, and the following appearances were found:

A tumour occupied a considerable portion of the anterior part of the right hemisphere of the brain. When the dura mater was removed, this tumour rose considerably higher than the surrounding parts, and it also pressed on the right ventricle. The blood-vessels of that part of the pia mater which lay over the surface of the tumour were almost obliterated. The medullary substance of the brain around the tumour was very soft; but the tumour itself, and more especially a central part, of about the size of a hazel nut, was of very firm texture. In the left ventricle there was about a table-spoonful of water; the other parts of the brain were in their natural state.

Case XI.—T. H., aged 30, on his admission into St. Bartholomew's Hospital, October 13, 1814, complained of an excruciating pain in his head, which he had suffered at intervals for six weeks; but within the last few days it had become almost constant. He stated, that the affection had first attacked him after working hard in a hay-field, which produced violent perspiration; that after this he had severe pain in his limbs; and, still further, that it had been much increased by bathing in the sea when he was heated. The violence of the pain compelled him to the utterance of a most distressing moaning sound, and, when sitting upright, he was scarcely able to hold up his head without support. It was situated across the forehead, and stretched round over the ear of the left more than that of the right side; but it seemed to have varied its situation, and at times to have occupied the right side also, and gone down the neck. It commonly increased in the night, and deprived him almost entirely of sleep; and frequently, but not constantly, it became throbbing with great violence. His sight was much affected, and he could not see so as to read ordinary print: the power of the left eye was much less than that of the right; its pupil was also more dilated, and it appeared half closed. The face seemed to be rather drawn to the left side, but there was no other appearance of paralysis, none of convulsive affection of any sort. The bowels were somewhat costive; the pulse was 86, weak and oppressed. Cupping and blistering had been previously employed without any relief. I directed the immediate administration of a purgative medicine, that the temporal artery should be opened, and that the quantity of blood drawn should be regulated according to his feelings and strength. He seemed to be relieved considerably by the bleeding; and it happened that the artery opened again four times in the two following days, with the loss altogether of a very large quantity; at last, however, it was secured by ligature. The pain afterwards seemed to exacerbate, chiefly at night, through which he became exceedingly restless, and was described as being delirious from its violence; but it abated during during the day, and his mind was at all times perfect when I saw him in the morning, though latterly he seemed unwilling to make the exertion of talking. The pupils became gradually less susceptible of the action of light, and more dilated, and the sight became more and more dim, until it was wholly lost. The bowels at all times acted readily, and latterly he passed his urine and fæces involuntarily. The pulse never rose above 96; usually it was nearer to its other extreme, which was 64. On November 3, he suffered a sudden attack of strong apoplectic symptoms, with a purple loaded countenance and stertorous breathing. On this account blood was drawn from the arm, which was firm, buffy, and cupped: it produced only a slight relief from the load, and he died November 5.

When the head was examined on the next day, the following appearances were exhibited. The membranes covering the brain were in a healthy state. The veins leading to the longitudinal sinus were more distended with blood than ordinary. The convolutions forming the superior and lateral parts of the hemispheres, with the intervening sulci, were but indistinctly manifest, their surface being smooth, and having a flattened appearance. From the inferior part of the anterior lobe of the left hemisphere, a mass of firm substance projected: it was of about the bulk of a large walnut, and, when cut into, resembled in appearance a large absorbent gland; it was embedded in diseased and softened medullary substance of the brain. The external surface of this hemisphere had given to the touch the sensation of a flaccid bag containing fluid; and the greatest part of its medullary matter was reduced to the state of a pulpy fluid, and was of a very light brown colour. In the part which more immediately surrounded the altered substance, the surface appeared rough, as if in a state of ulceration. The diseased appearance was confined to the medullary substance, and did not extend to the cortical. A small quantity of aqueous fluid was contained in the lateral ventricles.

Case XII.—D. M. G., a sailor, aged 48, was admitted into St. Bartholomew's Hospital, Feb. 16, on account of hæmoptysis, which had existed for more than three weeks, with slight pain in the side. The blood had been coughed up three or four times daily, and was often not less in quantity than half a pint. It varied in its appearance; sometimes it was florid and frothy, at others dark and clotted, and at others again mixed with yellowish matter resembling pus. His pulse was 112, and feeble, and he appeared pale and emaciated. His bowels acted regularly, and their discharges were natural. His tongue was slightly whitish and dry. I directed the infusion of roses in mint-water, with ten grains of alum, and one fluid drachm of compound tincture of camphor, to be taken every six hours; and that he should be kept quiet, and live upon a milk diet. The blood still flowed, but in less quantities, it was florid and frothy; the cough and pain continued, and he seemed to be purged by the medicine. He then took one grain of superacetate of lead, one grain of fox-glove, and half a grain grain of opium, in a pill, every four hours, with a mucilaginous mixture; and for a fortnight afterwards he passed no fresh blood, and his bowels became regular: the cough, however, and pain in the side were increased rather than diminished, and the latter was easier when he lay upon it. He did not gain flesh or strength, and his pulse was never below 108. On Feb. 27 he had a violent attack of cough, when he brought up a large quantity of blood mixed with pus; he still complained of pain, his nights were described as restless, and his head as wandering, though there was no appearance of delirium in the mornings. He took the infusion of roses in mint water, and forty minims of tincture of opium at night. After this, for the last three weeks of his life, he coughed and expectorated much less, and never brought up any quantity of blood; but his restlessness and disturbance of mind increased so much, as occasionally to render restraint necessary. This violence was chiefly in the night, for in the day time he usually lay in a dozing comatoze state, with eyes half closed, and pulse scarcely perceptible; sometimes, however, groaning dismally, so that I considered him as in a dying state, and rather exhibited comfortable nourishment, than insisted upon medicine, which he much disliked. He never seemed wholly to lose his senses, but was aware of what he said, though it was wild and nonsensical; and he asked chiefly for wine, not for any other nourishment. He sometimes passed his urine and fæces in bed, but he was conscious of it, and immediately desired its removal; and he sometimes threatened that he would do the same wilfully, if he was not assisted at the moment he desired. His bowels continued to act regularly, and their discharge was natural. He never expressed the existence of any pain in his head at any time. He died on March 19th.

After death, his chest was examined, and the right bag of the pleura contained a large quantity of pus, which was confined to the anterior part, and retained posteriorly by adhesion between the two pleuræ; the lung itself was almost obliterated, and occupied but a small part of the cavity. The left side of the thorax was sound. The liver was much enlarged, and of diseased structure. But the reason why I relate the case arose from an accidental examination of the head, which was made by Mr. Stanley, when the following appearances were found.

Beneath the anterior part of the corpus callosum, in the centre of the cerebrum, there was formed a cavity of sufficient size to hold a large wallnut, containing an admixture of thick pus and clots of blood; the surrounding cerebral substance being softened, and much altered from its natural texture. The commencement of the disease appeared to have been in the right hemisphere, near the lateral ventricle of the same side, into which the projection of the cerebral substance, containing the pus, had taken place. The cavity of the abscess was, however, distinct from that of the ventricle.

As, in some of the preceding cases, I have spoken of the abstraction of blood from the temporal artery, I think myself justified in in the further recommendation of this operation above all other modes, where, in diseases of the head, such abstraction is deemed necessary. It is easily performed, so much so, that in a case of great emergency, I once even ventured to undertake it myself; and any subsequent hæmorrhage from the artery which has sometimes occurred, as the preceding cases shew, may be easily subdued at any rate by ligature, or more easily by the complete division of the vessel. For the purpose of strengthening this opinion, I shall record one other case in which the relief it afforded was strikingly exemplified.

Case XIII.—A gentleman, past the middle age, had for some time laboured under slight but uniform aberrations of mind, but he was cheerful in his disposition, full of affection for his family, and harmless in his conduct. During this state he had more than once suffered a slight epileptic attack, which had soon passed away without more inconvenience. I was called to him on account of a sudden fit which had then lasted for some hours, and had not been diminished by ordinary venæsection, or by purgative medicines, although the latter had operated, and produced their full effect. His countenance was loaded and dark, his eyes were starting, his breathing was loudly stertorous, and his mouth covered with foam. He was wholly insensible to external applications, and appeared to be verging fast towards dissolution. His pulse, however, was quick, and so full and hard, that I was induced to urge evacuations to a greater extent. The temporal artery was upon this principle opened, and successfully, for the blood was thrown out from it in a full stream. It was thought that in so desperate a state the evacuation should not be carried too far, and therefore it was determined to allow its continuance until the pulse should begin to fail. Rather more than two pint basins were taken before any such effect was produced, and I left the patient with very little hope of advantage from the treatment adopted, or indeed from any other mode; nor was I a little surprised on the day following to find him sitting in his drawing-room, rather clearer in his intellect than usual, but without any knowledge of what had passed, and as well in his bodily health, except some feeling of weakness, as he had been for a considerable time. Some months afterwards a similar attack proved fatal; and I have to regret, that permission was not granted by his friends for an examination of the head.

I shall here terminate this gloomy catalogue of incurable disease. In offering it to the attention of the College, I have only hoped to connect some important morbid alterations of structure with their previous symptoms, and thus to contribute to some future general account of diseases of the brain. Such as they are, I have drawn them from notes made at the moment; and, as I hope and believe, faithfully. All of them have been under the notice of other practitioners also, whose names would stamp them with additional credit, but I have thought that, by omitting these, I should better avoid the attachment of them to individuals, which, where the concurrence of their surviving friends cannot well be asked, is to to my mind a proper delicacy; for the same reason also I have inserted no dates beyond what are necessary. Some of the diseased parts have been preserved; but I do not think they would have been more clearly illustrated by plates, than by a verbal description.

I shall shortly recapitulate the appearances recorded, with a reference to the cases. 1. A healthy state of brain, after stupor, insensibility, and convulsion. 2. Effusion of blood, with an instantaneous extinction of life. 3. A loaded state of the blood-vessels of the membranes, and an effusion of coloured fluid into the ventricles. 4. A strong and distinct adventitious membrane, covering the right hemisphere of the brain. 5. Caries of the temporal bone, with an effusion of pus and coagulable lymph under the dura mater of the right side. 6. Ulceration in the anterior lobe of each hemisphere of the brain, with aqueous effusion into the ventricles. 12. Ulceration in the brain. 7, 8, 9, 10, 11, 13. Tumours in the brain, of various structures, and in different situations. 14. A state of apoplexy, speedily removed by arteriotomy.

---

The subject of *Elephantiasis*, or Arabian leprosy, having engrossed much attention from the faculty since the cases described in the Medico-Chirurgical Transactions and in the Medical Transactions, (see our last Number,) we trust the following will not be found uninteresting; it is copied from a black-letter book in the possession of Sir Joseph Banks, published in the year 1541.

The title of the book is, "¶ The Questyonary of Cyrurgyens, with the Formulary of lytell Guydo in Cyrurgie, with the Spectacles of Cyrurgyens newly added, with the Fourth Boke of the Terapentyke,* or Methode curatyfe of Claude Galyen Prynce of Physyciens, with a syngular Treaty of the Cure of Ulceres, newely enprynted at London, by me Robert Wyer, and be for to sell in Poules Churcheyarde, at the sygne of Judyth. Cum privilegio ad imprimendum solum."

The chapter is superscribed.

"¶ The Manner to examyne Lazares; and to approue Lepry Meselry; after the Myndes of Doctours."

"As Galyen wytnesseth it is greate iniury be it done to man or woman to departe & put away theym that be nat infecte w[t] lepry, nor touched with meselry, & nat beynge lazares. And also it is greate danger to supporte, haunte, or be with suche as are stryken, or dyseased therwith; for it is a contagyous and dangerous malady. And therfore they that ought to iudge and approue them

---

* Copied correctly; but probably an error in the press for terapeutic, that is, *therapeutic*. It appears, however, by other parts of the book, that the *n* was sometimes used purposely where the Greek etymology would require the *u*, and where the latter is at this time used.

4 shulde shulde ryght dylygently beholde theym & considre the vnyuoke [unequivocal] sygnes and equyuokes also. And nat for one onely token gyue theyr sentences; but by many conuenaunces, and specyally vnyuokes. ¶ Fyrste than whan that the approuers come or cal them dyseased to theyr presence for to examyne them, they ought to conforte them with holsome wordes, and tel them that the sayd dysease is penaũce salutary for the saluacion of theyr soules, and byd them to take it pacyently. And that they feare nat to saye the trouth; for yf they were founde lazares it shuld be theyr purgatory in this worlde. For albeit that they were refused of the worlde; yet they were and be chosen of God, &c. And than cause them to swere to say the trouth; and enquyre of them suche thynges as foloweth. ¶ Secondly the examyners ought to enquyre of theym by the prymatyfe causes of lepry. And fyrste enquyre of them yf there were any of his lygnage that he knewe to be lazares; and specyally theyr faders or moders; for by any other of theyr kynred they ought nat to be lazares; but yf it were by some constellation that influed equally upon a kynred; and specyally on them that dwelt togyder, and haue one selfe maner of lyuynge; as me se oftentymes by the tyme of pestylence, yf any of a kynred be stryken or enfect; y^t also many other as bretherne, and cosyns, or other parentes soone after are stryken, & yet or they haue be borne. For as Auycen [Avicenna] sayeth in his seconde treatyse the fyrste ten of the fourth of his Canon in the fyrste chapyter of rottennes. The fyrste cause of rottennes is meates; and the nourysshynge that is of euyl qualytees. And for that cause yf a chylde be nourysshed of a woman corrupte and infecte in her humours ought also to be infect. And nat all onely yf the mother be a lepresse; but let us beholde also y^t for the sayd cause by experyence that they beyng conceyued in the tyme that y^e woman hath her floures; and that she be nat clene that scantly the chylde scapeth lepry, or to be scalled, or tached with suche infecte dyseases; or that he bere some tache upon hym. Also yf the father were fecte [infected] and in the mater wherof he is composed. For as Galyen sayeth in the fyrste particle of the efforysmes of Ypocras upon this canon Et qui crescunt. ¶ The thynges that are dissolued of an other thynge necessaryly extendeth of the nature of the thynge wherof they ar dissolued. Than ought ye to enquyre yf he hath had y^e company of any lepresse woman. And yf any lazare had medled with her afore hym and lately, bycause of the infect mater and contagyous fylth that she hadde receyued of hym. It is to be noted that a woman is nat so dangerous to be a lepresse to habyte with a lazare, as it shulde be a man to habyte with a lazarous woman, or with one that hath habited newly w^t a lazare. For all infections remayne in the matryce of the woman, unto the tyme that they be pourged by theyr floures & clensed; whiche a man can nat do, bycause he hath no receptacle where to holde the sayd imundycytees. ¶ Than ye oughte to enquyre of hym yf he hath had the quartayne feuers; and howe longe syth. For howbeit (sayth Auycen in his fyrste ten of the fyrste boke of Canon)

Canon) the feuer quartayne delyuereth a man of euyl melancholyke diseases; and wyte yf he hath nat had the emorroydes, and syth whan-like reason; the emorroydes kepeth that he fall nat in to inconuenyence. ¶ Than enquyre hym of his dreames, and yf his dremes be nat terryble, and that he seeth blacke thynges, and deuyls: suche dreames betoken the melancolyke humour to haue domynyon wherby he is so enclyned. And wyt of hym how he is wont to lyue; as yf he hath used meates with stronge spyce and in great quantyte, and strong wynes, or garlyke, lekes, onyons, and colewortes, olde chese, gotes flesshe, of beares, of foxes, of mesylswyne, or salt meates, and of unclene fysshe all at one table, & yf he haue continued therwith. And also of all maner herbes, and such meates as brenne the blode, and holly consumeth it. Than aske yf he hath had great solycytudes, & chargeable thoughtes that hath dried hym made hym melancolyke. ¶ Than ye oughte to beholde and consydre in your selfe of what complexyon he is, as wel naturall as accydentall, for suppose that lepry be a colde disease by incineraciō of humours, yet Auycen sayth, the most ancyent cause of lepry is the euyl complexyon of the lyuer that is so hote and drye that it breneth the blode & melancolyeth it. ¶ After y[t] the pacient hath ben examyned upon the fyrste causes that dyspose a persone to be a lazare, he hought to be examyned and approued by the sygnes of lepry aswel equyuocalles as vnyuocalles, and are the sygnes that cōueneth onely in this dysease; and the equyuocal sygnes conueneth them in dyuers maladyes. ¶ Of the vnyuocall sygnes. Fyrste than in procedynge as it is sayd to the knowledge of the vnyuocal sygnes, in folowing the doctryne of Ypocras in the fyrste boke of y[e] pronostikes sayeng. Primo enim egri facie ꝑ notabis. Fyrste y[u] shalt note y[e] sygnes apperynge in the face for they are the truest; for all the sygnes vnyuocalles are holden there bicause y in y[e] face amonge al other mēbres of the persone is no greater nombre of spyrytes bicause of the. v. organes of knowlege y[t] is there. That are the hearyng, speakynge, seyng, smellyng, & felynge, & also it is the barest of flesshe; and therfore it is soonest altered of all y[e] other mēbres; & at this cause Gordon preserued a man at Montpyllier. x. yeres to be cast out, agaynst the intencyon of all other doctours there, because y[e] tokens appered nat in the face, & yet it dyd ouer all the other membres. ¶ Fyrste than begyne at the heyght of y[e] heed, & beholde his here & his browes, & plucke at them, & loke yf with y[e] rote they drawe any flesshe by the rottenes & corrupcyon of theyr flesshe. Such by defaute of nourysshynge is soone seen. Item fele w[t] thy fynger yf his browes be nat grauellous, and ful of graynes, bycause that in al lepry the vertue assymulatyfe befayleth. And for that cause whan y[e] nourysshing cometh to the mēbres they may nat assēble theym to the mēbres at all, and therfore they remayne grayny, the whiche thynge mounteth alway nexte the membres bare of flesshe as is the face. Than beholde his eyen yf they be rounde specyally to the domestyke partye. Also lykewyse yf his eares be rounde & thycke and rugged. Also yf his nosethyrlles be wyde outwarde narow win & gnawen. Also yf his lyppes & gumes are foule stynkyng and corroded. Also yf his voyce be horse, and as he speaketh in y^e nose. And also yf his brethe and sweate stynke, and all that cometh fro hym; and yf there apere any straytnes of breth as yf wolde querken [choak], and for that cause haue the most haunte. Also yf his loke be steyed and horryble in maner of monster. These sygnes be vnyuocalles that alwaye betoken lepry; whan they are all or the moste parte of theym with the equyuocalles as it shall appere, and such sygnes come in lepry by these causes as Auycen sayeth. The fyrste generacyon of lepry is in the entrayles, and for that cause the lunges and lyghtes be hurte, and the pype of the voyce assysteth it, and causeth them to speke as it were in the nose. And for the rotten and corrupte fumes y^t mounte vpwarde by the conductes of the brayne, and the heares lessen and fall for defaute of good fedynge. And they appere in the face and in the brest.

"¶ Of the equyuocalles tokens.

"The doctoures put. vj. tokens equyuocalles. The fyrste is hardnes and tuberosyte of the ioyntes outwarde as the armes, legges, handes, and fete, for the drye mater, that is stopped by melancoly. The seconde is a morfewe colour & derke [freckle] for the blacke melancholyke humoure that corrupteth the blode. The thyrde is fallynge of heare, spoken of in the vnyuokes. The fourth is wastyng of a brawne, [muscle], and chyefly of a poulce, [thumb], so that whan it is pynched it abydeth vpryght by the consuption of the sayd muscle. The fyfth is the insensybylyte of the rotten humours of the outwarde partes extremytees, spredde within them. The. vi. is blacke coperous skal and scabbe in the face, and sores on the body by rotten humours and corrupt, y^t stryue with y^e euyll fumosytees. The seuenth is graynes under y^e tongue, & behynde the eares, the causes are in the vnyuokes. The. viii. is breñynge and felynge of pryckynges ouer all the body. The. ix. is ruggyshnes of the skynne in maner of a goos, for the greate drythe of the blode and humours. And therfore they oughte to be vnclad & water caste on them, and loke yf it take and synke in the skyne by cause of theyr drythe; where it semeth that they are anoynted they seme so moche to be fat. The. x. that they be of yl rule, and are comonly begylers. The. xi. that they haue terryble dremes, as I said before. The. xii. y^t they haue weyke poulces. The. xiii. they haue whyte vryne, thyne, and asshy. The. xiiii. theyr blode is blacke and duskysshe, of leady colour, and sandye; & to se this it must be wasshen and streyned.

"¶ The maner to let them blode, and to wasshe and strayne it.

"Fyloyne sayth, that there must be a great openynge in the veyne whan they be letten blode bycause the thycke blode shuld nat remayne and the thynne onely come out. And whan it is drawen, consydre the substance and the colour yf it be so as is abouesayde, and than wasshe it, and passe it through a fayre whyte cloth, and than loke on the flesshe that abydeth in the cloute, and yf it be graueylous and troublous it is a great token. Otherwyse take salte and medle it in the blode, and yf it melte soone. Another way, take his vryne and vynegre, and loke yf they wyll myngle togyther. Yet do thus, put some of the blode in to a basyn full of water, and yf it go downe to the botome lyke meale it is a token that he is a lazare.

"¶ Then good Cyrurgyen do nat as a folysshe iuge that forthwith gyueth his sentence; but fyrste or thou gyue it preferre God before thyne eyes; and consydre dylygently the vnyuocal sygnes and the equyuocalles, and se yf they agree, but yet neyther iudge a man to be lazarous by the equyuocalles, nor for one or two of the vnyuocalles, nor by the least of the pryncypalles; but there as the vnyuocalles in all or in the moste parte, and of the pryncypalles accorde with y[e] equyuocalles of the moste parte, and of the pryncypalles.

"Finis. ¶ Thus endeth the maner for to examyne lazares, and to approue their diseases after the intencyon of doctours."

The perusal of the above will at once shew that the tokens have been copied from the various writers who have followed Aretæus without any alteration, and with very few omissions.

Rymer's Fœdera contains, it is said, the only instance in which the disease is judicially described; and here the subject was not found with the tokens.* If any of our readers can produce an instance of any record in which they were discovered on an individual native of Britain, it might lead to a proof that this formidable disease was frequent in these northern regions, though at this time it is only found in emigrants from the south.

---

### CRITICAL ANALYSIS

### *OF RECENT PUBLICATIONS*

IN THE

* See Adams on Epidemics, p. 110.

